# Ocular Immune-Related Adverse Events Associated With Immune Checkpoint Inhibitors in Lung Cancer

**DOI:** 10.3389/fimmu.2021.701951

**Published:** 2021-08-24

**Authors:** Lin Zhou, Xin Wei

**Affiliations:** Department of Ophthalmology, West China Hospital, Sichuan University, Chengdu, China

**Keywords:** ocular immune-related adverse events, immune checkpoint inhibitors, lung cancer, ophthalmoplegia, uveitis, dry eye

## Abstract

Immune checkpoint inhibitors (ICIs) are novel immunotherapy-based drugs that have become increasingly popular in the treatment of lung cancer. Researchers have recognized ocular immune-related adverse events (irAEs) secondary to ICIs because of their vision-threatening characteristics. However, they are incompletely characterized and no studies have reported the ICI-related ocular irAEs in lung cancer. Therefore, we aimed to comprehensively illustrate the clinical characteristics, contributory factors, diagnosis, and management of ICI-related ocular irAEs in lung cancer, based on previously reported 79 patients. Ophthalmoplegia (40.51%), uveitis (20.25%), and dry eye (17.72%) were the most common ICI-related ocular irAEs in lung cancer. Ptosis was the most common (36.71%) and the highest mortality (23.33%) of ophthalmoplegia. Patients in Asia and patients who underwent combination therapy with programmed cell death-1 and cytotoxic T-lymphocyte-associated antigen 4 inhibitors demonstrated significantly higher frequency of ophthalmoplegia than other ocular irAEs. Most ICI-related ophthalmoplegia and uveitis in lung cancer were observed in the first 10 weeks following the initiation of ICIs. Furthermore, the onset time of dry eye and other ocular irAEs was much longer. In addition, 92.31% of the patients with ocular irAEs other than ophthalmoplegia could be remised. In conclusion, ocular irAEs secondary to ICIs in lung cancer are non-negligible, particularly ophthalmoplegia. Ethnicity and the type of ICIs play important roles in the distribution of ocular irAEs. ICI-related ophthalmoplegia in lung cancer presented with early onset and worse prognosis features, thus necessitating further attention.

## Introduction

Lung cancer is diagnosed in approximately two million people (11.6% of the total cancer cases), and is a leading cause of cancer death worldwide (1–3). Based on the histologic subtypes, lung cancer has been classified as large cell carcinoma, squamous carcinoma, and adenocarcinoma (NSCLC, non-small cell carcinoma), and small cell lung cancer. With the identification of molecular mechanisms by which cancerous cells evade T cell-mediated cytotoxic damage, immunotherapy has been considered as an effective treatment for patients with lung cancer (4–6).

Immune system plays an important role in monitoring and destructing cancer cells. However, this natural defense can be evaded by tumor cells and the upregulation of key immune checkpoints could increase the tolerance. Antitumor immunity may be blocked by suppression through the activation of immune checkpoints, including the cytotoxic T-lymphocyte-associated antigen 4 (CTLA-4) and programmed cell death-1 protein (PD-1) pathways. Blocking the inhibitory molecular axis using monoclonal antibodies targeting PD-1 (nivolumab, pembrolizumab), PD-L1 (atezolizumab, avelumab, and durvalumab), or CTLA-4 (ipilimumab) can reactivate the effector and cytotoxic T cells to destroy the tumor cells (7, 8). Immune checkpoint inhibitors (ICIs) provide a long-lasting response to treatment in both at the early and late stage of lung cancer (9–11). It has been considered as the first choice of second-line therapy for advanced NSCLC and as first-line therapy (4, 12, 13).

Compared to the traditional therapy, ICIs can over-activate the non-specific the immune system, which could cause autoimmune toxicities known as immune-related adverse events (irAEs) (14–18). This in turn can affect any organ system, including the skin, heart, lungs, liver, kidneys, central nervous, gastrointestinal, endocrine, musculoskeletal, haematological, and ocular systems. The most common systemic irAEs include fatigue (26%–53%), skin pruritus (25%–35%), skin rash (1%–50%), lymphocytopenia (10%–49%), and abnormal liver function (1%–46%) (19). Following ICIs, the aforementioned irAEs may manifest as a wide variety of forms ranging from mild to severe (20), and vary based on the organ system and severity (21, 22). The prevalence of ICI-related pneumonitis is higher in NSCLC than in other tumor type, based on data from the Immuno-Cancer International Registry (23, 24). In addition, lung cancer is reportedly one of the most common tumor with ICI-related ocular irAEs (25).

Ocular irAEs following ICIs can cause a deterioration of the quality of life and exert an influence on the compliance of patients. Approximately 2.8-4.3% of the patients suffered ocular irAEs, based on the Food and Drug Administration (FDA) Adverse Event Reporting System pharmacovigilance database (26–28). However, no studies have comprehensively analyzed ocular irAEs in lung cancer following ICIs. We aim to evaluate uncommon and serious ICI-related ocular irAEs associated with lung cancer. Based on relevant literature on ocular irAEs in lung cancer, we intent to illustrate the epidemiology, clinical characteristics, contributory factors, diagnosis, and management of ICI-associated ocular side effects in lung cancer.

## Epidemiology of Ocular irAEs in Lung Cancer

Despite being infrequent, ocular irAEs can cause a deterioration of the quality of life and affect patient compliance. Initially, the incidence of ICI-related ocular irAEs was estimated to be approximately 0.4%-1% in patients with moderate-to-severe ocular irAEs (19, 29). Recently, three studies with large sample sizes have reported an incidence of 2.8-4.3% (26, 30, 31). In addition, the actual frequency of ocular irAEs following ICIs could be underestimated because of insufficient attention. The incidence of ICI-associated ocular complications may be higher in real-world practice.

Ocular side effects secondary to ICIs are immune-related, and can affect any part of the eye and orbit. The distribution and frequency vary in different ocular irAEs on ICIs. In 2018, uveitis and dry eye had been reported as the most frequent ICI-related ocular side effects. Ocular irAEs were reported in 2.80% patients in a cohort of 996 patients with ICIs reported in Mayo clinic (31). Dry eye was observed in 57.14% of the patients with ocular irAEs, followed by uveitis in 14.28% of the patients (31). In relation to ICI-associated ocular surface toxicity, dry eye, conjunctivitis, and keratitis were reportedly the most common irAEs in a previous review involving 29 studies (32). However, a systematic review on ipilimumab considered uveitis (4.3%) as the most common ocular irAE (27, 28). Anterior uveitis is the most common phenotype among all types of uveitis (30). Despite some reports on ophthalmoplegia, it is not considered as a common side effect (19).

**Figure 1:**
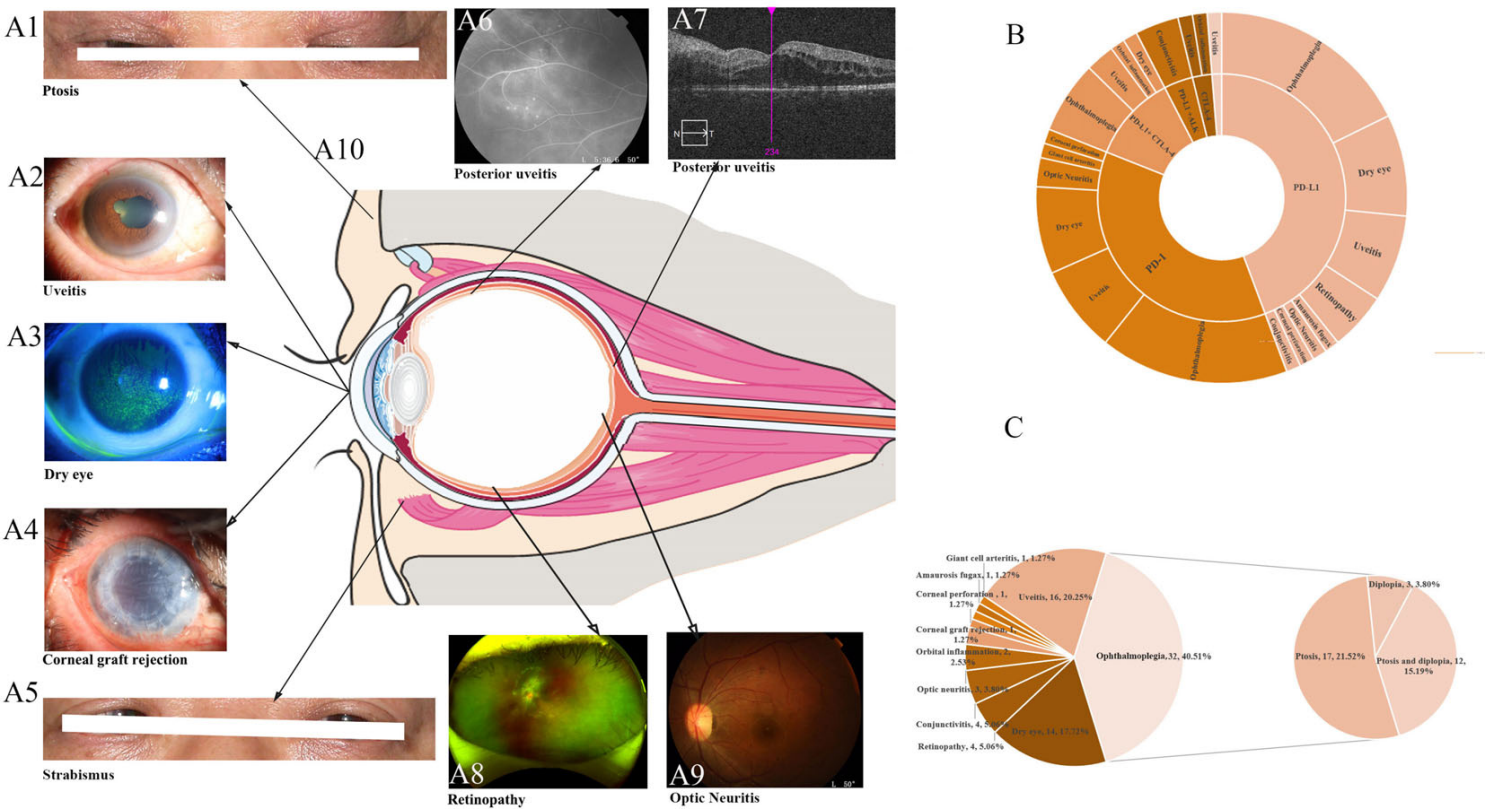
Clinical characteristics **(A)** and the distribution **(B, C)** of immune checkpoint inhibitor-mediated ocular side events. **(A)** The clinical characteristics of common ocular irAEs in lung cancer. **(B)** The distribution of ocular irAEs in different therapies. **(C)** A summary of all reported ocular irAEs in lung cancer following treatment with ICIs.

In this review, we summarized the reported ocular irAEs following ICIs in lung cancer by searching the PubMed database until April 2021 (25, 29, 33–86). The key words were a combination of ‘adverse events’, ‘lung’, and names of ICIs. We included studies describing ocular irAEs secondary to ICIs in lung cancer, and restricted the language of the selected literature to English. A total of 79 cases were detected, and the most frequently reported ocular irAEs following ICIs were ophthalmoplegia (40.51%), uveitis (20.25%), and dry eye (17.72%). In addition, we also identified retinopathy (5.06%), conjunctivitis (5.06%), optic neuritis (3.80%), and other frequent ocular irAEs, such as orbital inflammation (2.53%), amaurosis fugax (1.27%), giant cell arteritis (1.27%), corneal graft rejection (1.27%) and corneal perforation (1.27%) (**Figure 1**, **Tables 1**, **2**).

**Table 1:** Summary of reported ocular irAEs in lung cancer treated with immune checkpoint inhibitors.

Patient ID	Basic information									Treatment			Outcome		Ref
	Ocular irAEs	Age (years)	Genders	Country	Cancers Diagnosis	Onset (d)	ICIs	Target	Grade	ICIs	Treatment	Follow-up (d)	Ocular	Systemic	
1	Ophthalmoplegia	72	F	Japan	LC	NA	Pembrolizumab	PD-1	NA	NA	PSL (0.5 mg/kg), IVMP	NA	CCR	Alive	46
2	Ophthalmoplegia	77	F	Japan	NSCLC	48	Pembrolizumab	PD-1	NA	NA	NA	203	CCR	Alive	85
3	Uveitis	69	F	USA	SCLC	14	Ipilimumab and nivolumab	PD-L1+ CTLA-4	3	Stop	TS	60	CCR	Alive	73
4	Ophthalmoplegia	79	F	Belgium	LUAD	NA	Pembrolizumab	PD-1	NA	Stop	CHO-I, PSL	97	CCR	Alive	52
5	Uveitis	NA	NA	USA	NSCLC	NA	Pembrolizumab	PD-1	NA	NA	NA	NA	NA	NA	54
6	Uveitis	NA	NA	Japan	NSCLC	NA	pembrolizumab	PD-1	NA	NA	NA	NA	NA	NA	24
7	Dry eye	51	M	Spain	LC	90	Durvalumab	PD-L1	NA	NO	TS	NA	NA	NA	67
8	Uveitis	NA	NA	USA	NSCLC	NA	Avelumab	PD-L1	NA	NA	NA	NA	NA	NA	48
9	Conjunctivitis	NA	NA	Spain	NSCLC	NA	Nivolumab and Ceritinib	PD-L1 +ALK	NA	NA	NA	NA	NA	NA	48
10	Conjunctivitis	NA	NA	Spain	NSCLC	NA	Nivolumab and Ceritinib	PD-L1 +ALK	NA	NA	NA	NA	NA	NA	24
11	Dry eye	72	M	Spain	LC	60	Pembrolizumab	PD-1	NA	NO	NA	NA	NA	NA	24
12	Dry eye	58	M	Spain	LC	180	Pembrolizumab	PD-1	NA	NO	TS	NA	NA	NA	53
13	Uveitis	NA	NA	USA	NSCLC	NA	Pembrolizumab and CPB	PD-1+ chemotherapy	NA	NA	NA	NA	NA	NA	24
14	Dry eye	61	F	Spain	LC	300	Nivolumab	PD-L1	NA	NO	TS,	NA	NA	NA	24
15	Dry eye	64	M	Spain	LC	30	Durvalumab	PD-L1	NA	Stop	TS, PSL, IVMP, PE	NA	NA	NA	24
16	Dry eye	70	M	Spain	LC	540	Nivolumab	PD-L1	NA	Stop	TS	NA	NA	NA	24
17	Dry eye	71	M	Spain	LC	60	Nivolumab	PD-L1	NA	NO	TS	NA	NA	NA	68
18	Orbital inflammation	70	M	Italy	LUAD	30	Durvalumab and tremelimumab	PD-L1+ CTLA-4	NA	Stop	PSL (25 mg)	NA	NA	NA	65
19	Giant cell arteritis	88	F	USA	NSCLC	14	Pembrolizumab	PD-1	NA	NO	PSL	NA	NA	NA	38
20	Dry eye	50	F	Spain	LC	150	Pembrolizumab	PD-1	NA	NO	TS, PSL	NA	NA	NA	38
21	Dry eye	79	F	Spain	LC	30	Pembrolizumab	PD-1	NA	NO	TS, PSL, IVMP	NA	NA	NA	48
22	Conjunctivitis	NA	NA	Spain	NSCLC	NA	Nivolumab and Ceritinib	PD-L1 +ALK	NA	NA	NA	NA	NA	NA	24
23	Dry eye	68	F	Spain	LC	180	Nivolumab	PD-L1	NA	Stop	TS	NA	NA	NA	24
24	Dry eye	72	F	Spain	LC	210	Ipilimumab and nivolumab	PD-L1+ CTLA-4	NA	NO	TS, PSL	NA	NA	NA	67
25	Dry eye	NA	NA	USA	NSCLC	NA	Avelumab	PD-L1	NA	NA	NA	NA	NA	NA	24
26	Dry eye	71	M	Spain	LC	210	Pembrolizumab	PD-1	NA	Stop	TS	NA	NA	NA	69
27	Ophthalmoplegia	NA	NA	USA	SCLC	NA	Ipilimumab and nivolumab	PD-L1+ CTLA-4	NA	NA	NA	NA	NA	NA	56
28	Ophthalmoplegia	NA	NA	China	NSCLC	NA	Pembrolizumab	PD-1	NA	NA	NA	NA	NA	NA	41
29	Corneal graft rejection	58	F	France	NSCLC	126	Nivolumab	PD-L1	NA	Stop	TS, PSL, IVMP	30	Aggravation	Death	37
30	Ophthalmoplegia	57	M	China	LUSC	14	Ipilimumab and nivolumab	PD-L1+ CTLA-4	NA	NA	CHO-I, PSL (1mg/kg/d), IVMP	14	Remission	Alive (PD)	44
31	Orbital inflammation	68	F	USA	NSCLC	14	Ipilimumab	CT+2:73LA-4	NA	Stop	TS, PSL	7	Remission	Alive (PD)	44
32	Uveitis	54	F	USA	NSCLC	28	Ipilimumab	CTLA-4	3	NA	TS	42	Remission	Death	51
33	Ophthalmoplegia	65	M	Italy	LUSC	27	Nivolumab	PD-L1	NA	NA	CHO-I, PSL, IVMP	49	Aggravation	Death	36
34	Ophthalmoplegia	70	M	USA	SCLC	16	Ipilimumab and nivolumab	PD-L1+ CTLA-4	NA	Stop	PSL (90 mg), IVMP, PE	29	Aggravation	Death	76
35	Ophthalmoplegia	74	M	USA	LC	NA	Pembrolizumab	PD-1	NA	NA	CHO-I, PSL (10 mg), IVMP	28	Aggravation	Alive (PD)	25
36	Ophthalmoplegia	64	M	USA	NSCLC	NA	Durvalumab	PD-L1	NA	NA	PSL	NA	Aggravation	Death	37
37	Ophthalmoplegia	65	M	China	LUSC	53	Nivolumab	PD-L1	NA	NO	CHO-I, PSL (1mg/kg),	27	Aggravation	Alive (PD)	34
38	Ophthalmoplegia	76	F	Japan	LUAD	26	Nivolumab	PD-L1	NA	Stop	PSL (10mg), IVMP, PE	65	Remission	Death	74
39	Ophthalmoplegia	68	F	USA	NSCLC	70	Nivolumab	PD-L1	NA	Stop	CHO-I, PSL (60 mg)	18	Aggravation	Death	79
40	Ophthalmoplegia	61	M	France	NSCLC	NA	Nivolumab	PD-L1	NA	Stop	IVMP	77	Remission	Death	78
41	Uveitis	60	F	USA	LC	NA	Ipilimumab and nivolumab	PD-L1+ CTLA-4	NA	NO	PSL, immunosuppressive	84	Remission	Alive (PD)	50
42	Ophthalmoplegia	73	F	Japan	LUSC	140	Nivolumab	PD-L1	NA	NO	CHO-I, PSL (20 mg)	120	Aggravation	Alive (PD)	84
43	Dry eye	36	F	France	LC	39	Pembrolizumab	PD-1	NA	NA	TS, PSL (10 mg)	60	Remission	Alive	57
44	Ophthalmoplegia	73	M	Japan	LUAD	23	Pembrolizumab	PD-1	NA	NA	PSL (20 mg), IVMP	120	Remission	Alive	62
45	Corneal perforation	68	M	Belgium	LUAD	126	Pembrolizumab	PD-1	NA	Stop	TS, Surgery, PSL (32 mg)	30	Remission	Alive	47
46	Uveitis	71	M	Japan	LUSC	14	Pembrolizumab	PD-1	3	Stop	TS, PSL (70 mg)	21	Remission	Alive	81
47	Ophthalmoplegia	69	F	Japan	NSCLC	NA	Nivolumab	PD-L1	NA	NA	PSL, IVMP	36	Remission	Alive	66
48	Retinopathy	40	M	USA	NSCLC	13	Atezolizumab	PD-L1	NA	Stop	NA	21	Remission	Alive	86
49	Retinopathy	64	M	Spain	NSCLC	600	Durvalumab	PD-L1	NA	NO	PSL (30 mg), IVMP	60	Remission	Alive	87
50	Uveitis	53	M	USA	NSCLC	19	Nivolumab	PD-L1	3	Stop	Surgery, PSL (1mg/kg)	9	Remission	Alive	80
51	Uveitis	68	M	USA	LUAD	NA	Atezolizumab	PD-L1	4	Stop	NA	90	Remission	Alive	35
52	Ophthalmoplegia	65	M	USA	NSCLC	14	Nivolumab	PD-L1	NA	Stop	CHO-I,	42	Remission	Alive	45
53	Uveitis	54	F	Japan	LC	NA	Nivolumab	PD-L1	3	NO	TS, PSL (30mg)	135	Remission	Alive	33
54	Optic Neuritis	76	M	Spain	NSCLC	72	pembrolizumab	PD-1	NA	NA	PSL(0.5mg/Kg/day), IVMP	21	Remission	Alive	66
55	Retinopathy	50	M	USA	NSCLC	13	Atezolizumab	PD-L1	NA	Stop	NA	21	Remission	Alive	30
56	Amaurosis fugax	84	M	USA	NSCLC	NA	Nivolumab	PD-L1	NA	NA	NA	NA	Remission	Alive	43
57	Uveitis	61	F	USA	NSCLC	60	Durvalumab	PD-L1	4	NO	TS	30	Remission	Alive	29
58	Uveitis	63	F	France	NSCLC	36	Nivolumab	PD-L1	3	NA	TS	42	Remission	Alive	63
59	Uveitis	61	M	Japan	NSCLC	63	Pembrolizumab	PD-1	NA	Stop	PSL	NA	Remission	Alive	42
60	Retinopathy	64	F	USA	LUAD	7	Nivolumab	PD-L1	NA	Stop	PSL (60mg)	21	Remission	Alive	46
61	Ophthalmoplegia	53	M	Japan	NSCLC	27	Nivolumab	PD-L1	NA	NA	PSL (30mg), IVMP	49	Remission	Alive	60
62	Ophthalmoplegia	83	M	Japan	LUSC	38	Pembrolizumab	PD-1	NA	NA	CHO-I,PSL (20 mg)	51	Remission	Alive	38
63	Ophthalmoplegia	65	M	Espada	LUAD	–	Nivolumab	PD-L1	NA	Stop	CHO-I	NA	Remission	Alive	46
64	Ophthalmoplegia	46	F	Japan	NSCLC	30	Nivolumab	PD-L1	NA	NA	NA	14	Remission	Alive	55
65	Ophthalmoplegia	77	F	Japan	LUAD	49	Pembrolizumab	PD-1	NA	NA	PSL, IVMP	209	Remission	Alive	25
66	Optic Neuritis	74	M	USA	NSCLC	NA	Pembrolizumab	PD-1	NA	Stop	NA	NA	Remission	Alive	46
67	Ophthalmoplegia	78	M	Japan	NSCLC	38	Pembrolizumab	PD-1	NA	NA	PSL (80mg), IVMP	91	Remission	Alive	46
68	Ophthalmoplegia	83	M	Japan	NSCLC	28	Pembrolizumab	PD-1	NA	NA	PSL (20mg)	42	Remission	Alive	71
69	Ophthalmoplegia	66	M	China	LUAD	21	Sintilimab	PD-1	NA	NA	CHO-I, PSL (60 mg), IVMP, IVIg, PE	90	Remission	Alive	61
70	Uveitis	55	F	USA	LC	42	Pembrolizumab	PD-1	2	NA	TS	42	Remission	Alive	83
71	Conjunctivitis	67	M	Switzerland	LUAD	182	Nivolumab	PD-L1	NA	NO	TS	NA	Remission	Alive	82
72	Optic Neuritis	64	M	Japan	NSCLC	365	Pemetrexed	PD-L1	NA	NA	PSL (30 mg), IVMP	3	Remission	Alive	70
73	Ophthalmoplegia	66	M	Australia	LUAD	49	Durvalumab	PD-L1	NA	Stop	CHO-I, PSL (60 mg), IVIg	14	Remission	Alive	75
74	Ophthalmoplegia	66	M	Spain	LC	28	Ipilimumab and nivolumab	PD-L1+ CTLA-4	NA	Stop	CHO-I, IVMP	28	Remission	Alive	46
75	Ophthalmoplegia	73	M	Japan	NSCLC	33	Pembrolizumab	PD-1	NA	NA	PSL (20mg), IVMP	98	Remission	Alive	39
76	Ophthalmoplegia	68	M	USA	NSCLC	30	Durvalumab and tremelimumab	PD-L1+ CTLA-4	NA	NO	PSL (60mg)	30	Remission	Alive	58
77	Uveitis	71	M	Japan	NSCLC	NA	Pembrolizumab	PD-1	3	NA	PSL (30 mg), IVMP	84	Remission	Alive	47
78	Ophthalmoplegia	76	M	South Korea	NSCLC	NA	Nivolumab	PD-L1	NA	NO	CHO-I, PSL, IVMP	30	Remission	Alive	64
79	Ophthalmoplegia	63	F	USA	LUAD	28	Pembrolizumab	PD-1	NA	NA	CHO-I, PSL, IVIg	NA	Remission	Alive	75

NSCLC, n-small cell lung cancer; SCLC, small cell lung cancer; LUAD, Lung adenocarcinoma; LUSC, Lung Squamous Cell Cancer; NA, t available; IVMP, Intravenous methylprednisolone; IVig, Intravenous methylprednisolone. CHO-I, cholinesterase inhibitor; TS, Topical steroid; RTD, artificial tear drops; PE, Plasma exchange; CCR, Complete clinical recovery; Ref, reference; PD, Progressive disease; NO, continue.

**Table 2:** Comparison of the ophthalmoplegia, uveitis and other ocular irAEs secondary to ICIs in lung cancer.

	Total (%)	A	B	C	D	P value
		Ophthalmoplegia (%)	Uveitis (%)	Dry eye (%)	Others (%)	Ophthalmoplegia *VS* other irAEs (Uveitis, Dry eye and Others)
NO.	79(100.00)	32(40.51)	16 (20.25)	14 (17.72)	17 (21.52)	
Age	66.22 ± 9.95	69.03 ± 8.22	61.67 ± 6.52	63.31 ± 11.40	66.79 ± 11.94	
Gender						(Male *VS* Female)
Male	42 (53.16)	20 (62.5)	5 (31.25)	7 (50)	10 (58.82)	8.00E-02
Female	27 (34.18)	10 (31.25)	7 (43.75)	6 (42.86)	4 (23.53)	
NA	10 (12.66)	2 (6.25)	4 (25)	1 (7.14)	3 (17.65)	
Age						(≤65 *VS* >65)
≤65	30 (37.97)	10 (31.25)	8 (50)	6 (42.86)	6 (35.29)	2.46E-02
>65	39 (49.37)	20 (62.5)	4 (25)	7 (50)	8 (47.06)	
NA	10 (12.66)	2 (6.25)	4 (25)	1 (7.14)	3 (17.65)	
Onset(d)	86.31 ± 119.81	37.73 ± 26.10	34.50 ± 18.18	159.92 ± 136.79	130.17 ± 136.79	
Ethnicity						(Caucasian *VS* Asian)
Caucasian	59 (74.68)	18 (56.25)	11 (68.75)	14 (100)	16 (94.12)	1.21E-06
Asian	20 (25.32)	14 (43.75)	5 (31.25)	0 (0)	1 (5.88)	
Unilateral or Bilateral						(Unilateral *VS* Bilateral)
Unilateral	28 (35.44)	11 (34.38)	10 (62.5)	0 (0)	7 (41.18)	1.46E-01
Bilateral	16 (20.25)	8 (25.00)	2 (12.5)	0 (0)	6 (35.29)	
NA	35 (44.3)	13 (40.63)	4 (25.00)	14 (100)	4 (23.53)	
ICIs						(PD-1 *VS* PDL-1 *VS* PD-L1+CTLA4)
PD-1	29 (36.71)	13 (40.63)	6 (37.5)	6 (42.86)	4 (23.53)	2.07E-01
PD-L1	35 (44.3)	14 (43.75)	6 (37.5)	7 (50)	8 (47.06)	
PD-L1+CTLA4	9 (11.39)	5 (15.63)	2 (12.5)	1 (7.14)	1 (5.88)	
Others	6 (7.59)	0 (0)	2 (12.5)	0 (0)	4 (23.53)	
Outcome(Ocular)						(Aggravation *VS* Remission)
Aggravation	9 (11.39)	7 (21.88)	0 (0)	1 (7.14)	1 (5.88)	3.98E-04
Remission	47 (59.49)	23 (71.88)	12 (75)	1 (7.14)	11 (64.71)	
NA	23 (29.11)	2 (6.25)	4 (25)	12 (85.71)	5 (29.41)	
Survival state						(Death *VS* Alive)
Death	8 (10.13)	7 (21.88)	1 (6.25)	0 (0)	0 (0)	1.82E-09
Alive	48 (60.76)	23 (71.88)	11 (68.75)	2 (14.29)	12 (70.59)	
NA	23 (29.11)	2 (6.25)	4 (25)	12 (85.71)	5 (29.41)	

NO., number; d, days; NA, not available; d, days; NS, no significant difference; CCR, Complete clinical recovery.

## Clinical Characteristic of Ocular irAEs in Lung Cancer With ICIs

### The Onset Time of Ocular irAEs in Lung Cancer

**Figure 2:**
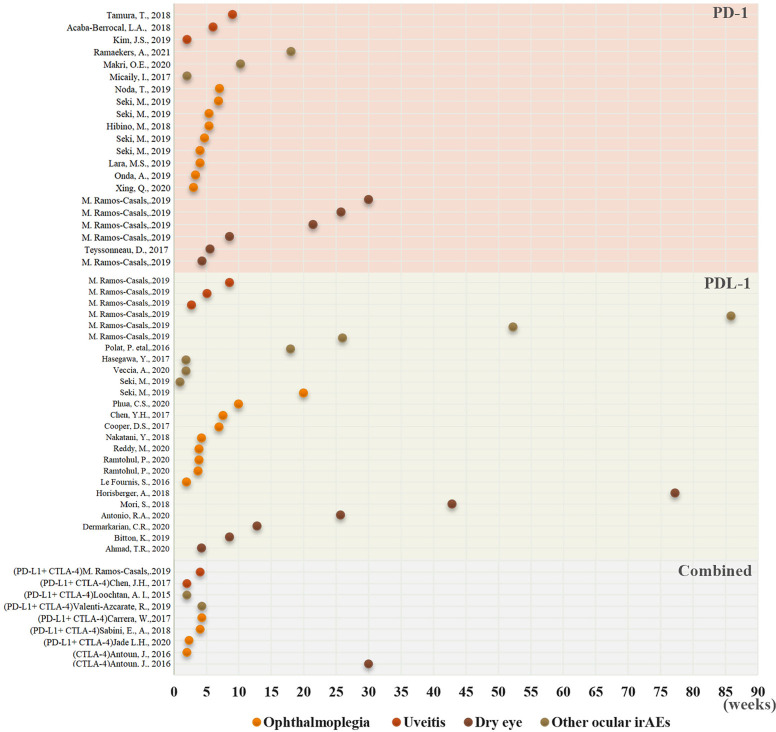
The onset time of the distribution of different ocular irAEs in lung cancer following ICI use. The onset time of ocular irAE detection has been recorded as a dot. Yellow, ophthalmoplegia; dark yellow, uveitis; brown, dry eye; and darkgray, other ocular irAEs.

**Figure 3:**
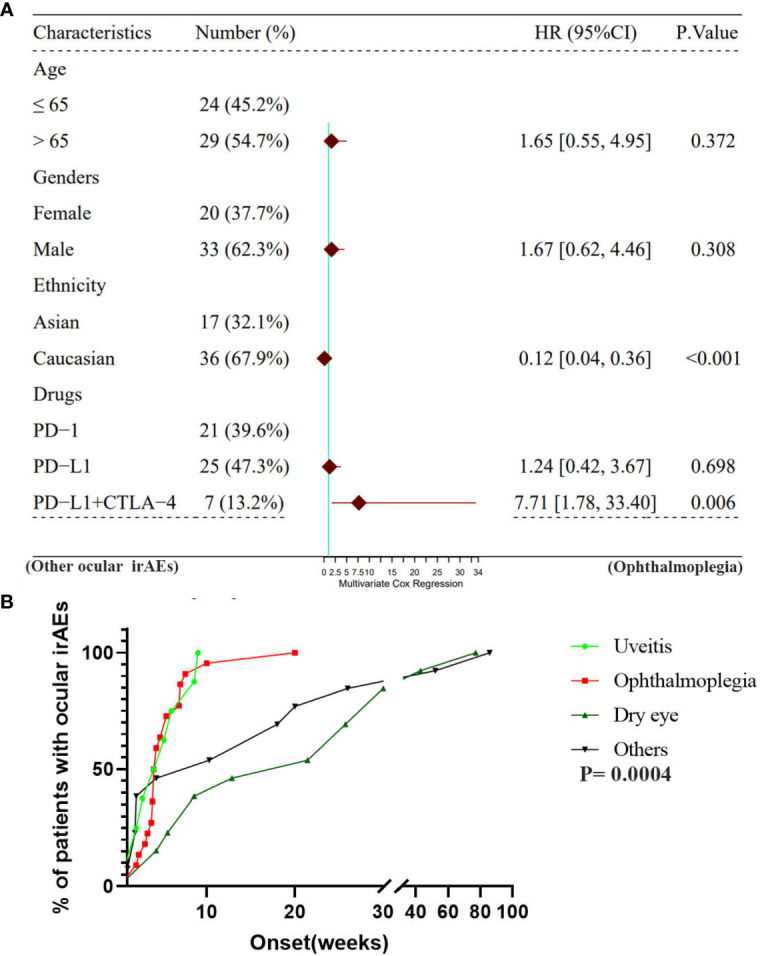
**(A)** A multivariate cox regression analysis for the ocular irAEs among age, gender, ethnicity, ICIs drugs. **(B)** A comparison of the onset time of ocular irAEs among ophthalmoplegia, uveitis, dry eye, and other ocular irAEs.

The mean time to the onset of ocular irAEs in lung cancer was approximately 35 days, and the overall time ranged from 28.0–111.5 days (19, 87). Moreover, 73% of the patients developed ocular irAEs within 60 days following ICIs initiation. While intraocular inflammation was detected after a median 9 weeks, 83.6%-91.67% of the patients were diagnosed with uveitis within 6 months (median 63 days) (28, 88). Ophthalmoplegia was diagnosed at a median onset of 35 days. According to recent reviews on ocular adverse events, the average onset time of ophthalmoplegia was approximately 6 weeks after ICIs initiation (range 2–12 weeks) (19, 89–91). The median interval between the onset of ICIs use and the diagnosis of dry eye was 6.5 months in 26 patients secondary to ICIs (24). In this review, the average onset time of ocular irAEs in lung cancer was 57.28 days following ICIs (**Tables 1**, **2**, **Figures 2**, **3**). The average time was significantly shorter in patients with uveitis and ophthalmoplegia (32.22 days and 38.26 days, respectively) than those with other ocular irAEs (96.5 days) in lung cancer. More importantly, all ICI-related ophthalmoplegia and the majority of uveitis occurred in the first 10 weeks. However, the onset time of dry eye and other ocular irAEs was much longer (**Figures 2**, **3**). Furthermore, we did not detect a significant difference in the onset time of ocular irAEs in lung cancer among different ICIs, age, sex, and ethnicity (**Figure S1**).

### The Clinical Manifestation of Ocular irAEs in Lung Cancer

All of the ocular irAEs following ICIs were noninfectious and caused by the over-activate the immune system. **Table 3** summarizes the clinical characteristics and necessary examinations for different ocular irAEs. Ophthalmoplegia, uveitis and dry eye were the most common ocular irAEs secondary to ICIs in lung cancer have been described separately in detail as follows. Other ocular surface complications (conjunctivitis, corneal perforation, corneal graft rejection, retinopathy, optic neuritis, amaurosis fugax, giant cell arteritis and orbital inflammation) are also briefly discussed.

**Table 3:** The clinical characteristics, diagnosis, and treatment for ocular irAEs on ICIs therapy in lung cancer.

Ocular irAEs		Clinical characteristics	Diagnosis test	Treatment
Ophthalmoplegia		Ocular: ptosis, diplopia, blurred vision	1. Edrophonium test	1. Cholinesterase inhibitor
			2. The ice pack test	2. Systemic Corticosteroids
			3.Antibody assays (ACht Ab; Anti-MuSK Ab; LRP4	3. IVMP, IVig, PE, Stop ICIs when necessary
		Systemic: Difficulty in breathing, swallowing, chewing, walking, using arms or hands, or holding up head.	4.Neurophysiological tests (RNS and SfEMG)	
Uveitis	Anterior uveitis	Pain, redness, photophobia, blurred vision;	Slit-lamp examination	1. Topical Corticosteroids
		Anterior chamber cells and flare; keratic precipitates, posterior synechiae, iris nodules and cataract.		2. Topical mydriatics
	Intermediate uveitis	Floaters and blurred vision;	Slit-lamp examination; Anterior segment OCT	1. Topical Corticosteroids
		Vitreous cells, vitreous haze, ‘snowbanks’ (grey-white fibrovascular plaques).		2. Systemic Corticosteroids
	Posterior uveitis	‘Floaters’, blurred vision and blind spots;	Ophthalmoscopy; OCT; FFA	1. Topical corticosteroids
		Unifocal, or multifocal, generally white lesions.		2. Systemic corticosteroids
				3. Subconjunctival/Periocular corticosteroids
				4. IVMP, IVig, PE, Stop ICIs when necessary
	Panuveitis	All clinical characteristics of the anterior, intermediate and posterior uveitis	All diagnosis test of the anterior, intermediate and posterior uveitis	All diagnosis treatment of the anterior, intermediate and posterior uveitis
Dry eye		Eye dryness, eye redness, eye fatigue, photophobia, a sensation of burning, stinging or foreign body	Ocular: Tear film break-up time with fluorescein, Schirmer test, examination of the eyelid margins and meibomian gland orifices with expression of meibomian secretion	Artificial tears, Autologous serum eyedrops, Topical corticosteroid, Topical Cyclosporine A
			(For dry eye in Sjögren syndrome) Systemic: The Gum test, the unstimulated whole saliva, Saxon test, the labial salivary glands biopsy, and parotid glands biopsy	
Conjunctivitis		Red, itchy, watery, burning or stinging eye and foreign-body sensation	Slit-lamp microscope	1. Topical sodium hyaluronate, antihistamine eye drops
				2. Topical corticosteroids
Corneal perforation		Red eyes, severe pain, foreign-body sensation, tears, blurry vision, swollen eyelids	Slit-lamp microscope	1. Discontinue the ICIs
				2. Topical artificial tear drops, corticosteroids and cyclosporine;
		White spot on cornea, edema of cornea		3. IVMP, IVig, PE when necessary
Corneal graft rejection		Pain, redness, and decreased vision, conjunctival hyperemia, keratic precipitates, opacity and edema of corneal graft	Slit-lamp microscope	1. Discontinue the ICIs
				2. Topical artificial tear drops, corticosteroids and cyclosporine;
				3. IVMP, IVig, PE,cytotoxic agents, cyclosporin A when necessary
Retinopathy		Vision loss, scotomas, photopsia, nyctalopia	OCT, FFA, VF and electrophysiology	1. Topical Corticosteroids
				2. Systemic Corticosteroids
				3. IVMP, IVig, PE when necessary
Optic neuritis		Eye pain, vison loss, the loss of the visual field, flashing lights	Slit-lamp microscope, pupillary light reaction test, OCT, visual field test, visual evoked response	1. Systemic corticosteroids
				2. Subconjunctival/Periocular corticosteroids
				3. IVMP, IVig, PE when necessary
Amaurosis fugax		Transient visual loss	Comprehensive ocular examination and assessment of cardiovascular system (electrocardiogram, magnetic resonance angiography, blood test)	Control and treat potential vascular risk factors
Giant cell arteritis		Blurred vision, diplopia, amaurosis fugax and blindness	Ophthalmoscopy, FFA and ICGA are needed. Additionally, biopsy of the temporal artery, high-resolution color doppler ultrasound of the cranial and axillary arteries, MRI, CT scan	1. Systemic corticosteroids
		Headaches, scalp tenderness, jaw claudication, absent pulses and limb claudication		2. Subconjunctival/Periocular corticosteroids
				3. IVMP, IVig, PE, Stop ICIs when necessary
Orbital inflammation		Eye pain, proptosis, decreased visual acuity, and diplopia	Laboratory evaluation, Orbital ultrasound, Computed Tomography, and Magnetic Resonance Imaging	1. Systemic corticosteroids
				2. Nonspecific steroid-sparing agents(methotrexate, cyclosporin-A et al), biologic agents (infliximab, adalimumab and so on) and radiation therapy

OCT, Optical Coherence Tomography; VEP, Visual Evoked Potential; FFA, Fundus Fluorescein Angiography; UBM, Ultrasound Biomicroscopy; CT, Computed Tomographic Scans; TBUT, tear film break-up time with fluorescein; IVMP, Intravenous methylprednisolone; IVig, Intravenous methylprednisolone; PE, Plasma exchange.

#### Ophthalmoplegia in Lung Cancer Secondary to ICIs

Ophthalmoplegia is the dysfunction (weakness or paralysis) of one or more muscles that control eye movement. Ptosis is the earliest and most common manifestation of ophthalmoplegia, followed by diplopia and strabismus. In this review, 53.12% of patients with ophthalmoplegia suffered ptosis, 37.50% suffered ptosis with diplopia/strabismus. Only three patients (9.38%) complained of diplopia. Ptosis occurs when the upper eyelid droops over the eye, which in turn makes the affected eye appear smaller than normal eyes. The eyelid may droop just a little or completely covering the pupil (92). Moreover, it can be unilateral or bilateral. According to previous studies, ophthalmoplegia in lung cancer secondary to ICIs were accompanied by myasthenia gravis (MG) in all patients (25, 34–39, 46, 47, 49–51, 55–57, 60, 64, 69–76, 79, 81). Ptosis is the key manifestation of immune-related MG, and accounts for 75%–78.7% of ICI-induced MG (irMG) (93–96). Only 15% of ptosis continue to be isolated ocular complaints throughout the course of MG. MG is an autoimmune neuromuscular disease caused by antibodies directed against the postsynaptic muscle membrane. Moreover, it is reported as a life-threatening irAE with rapid deterioration shortly following ICI use (93, 97, 98). The most common reported manifestations of ICI-related MG are ptosis (75%), dyspnea (62%), limb weakness (55%), dysphagia (48%), and diplopia (42%) following ICI use (93). Severe muscle dysfunction with respiratory affectation, myocarditis, and/or myositis can also be detected in approximately two-thirds of individuals suffering from MG, and are the most fatal manifestations requiring mechanical support (89, 93). Approximately 20% of the individuals could die of MG upon an increase in respiratory dysfunction (99). In addition, the appearance of ophthalmoplegia caused by irMG can rapidly progress (96). Despite such patients with ptosis in ICI-related MG receiving discontinued ICIs and appropriate treatment with immunosuppression, their mortality rates are reportedly above 40% (100).

The high incidence of ophthalmoplegia in MG and the high mortality of life-threatening inhibitor-induced MG in lung cancer necessitate an increase in ophthalmoplegia vigilance. This will ensure the timely identification of irMG signs and early treatment, particularly in the early stages of irAEs. In this review, all recruited patients with ophthalmoplegia were diagnosed with MG in lung cancer following the use of ICIs. It could be unilateral (57.89%) or bilateral (42.11%), and the average onset time of ophthalmoplegia was 37.73 days following ICI initiation (**Table 2**). There were 66.67% men, and 66.67% patients were older than 65 years. Ptosis accounted for 90.63% of the patients with ICI-related ophthalmoplegia in lung cancer, followed by diplopia and strabismus.

It is difficult to make a definitive diagnosis of ophthalmoplegia in MG based on the clinical characteristics (101). However, electrophysiology and detectable antibodies could facilitate the diagnosis (102). The edrophonium test, ice pack test, antibody assays (acetylcholine receptor auto-antibodies; anti-muscle-specific tyrosine kinase auto-antibodies; low-density lipoprotein receptor-related protein 4), and neurophysiological tests (repetitive nerve stimulation and single-fibre electromyography) are the necessary examinations for the diagnosis of ptosis in MG (**Table 3**). Ophthalmoplegia in MG in lung cancer secondary to ICIs should be differentiated from other causes, which might also result in ptosis, including central disorders of ocular motility, congenital ptosis, inherited ptosis-associated syndrome, aponeurotic ptosis, and ptosis caused by local eye problems or muscles (103, 104).

#### Uveitis in Lung Cancer Secondary to ICIs

Uveitis describes a group of inflammatory diseases that produce swelling and destroy the uveal tract. The uveal tract consists of a pigmented, highly vascular, and loose fibrous tissue, prone to immune disorders. It can be divided into three anatomical regions as follows: anterior (involves the iris), intermediate (involves the vitreous humor), posterior (involves the choroid), and panuveitis (widespread involvement across anatomical regions) by the Standardisation of Uveitis Nomenclature Working Group. The aforementioned types of uveitis have varied clinical characteristics, diagnostic tests, and treatment (**Table 3**). Symptoms of pain, redness, photophobia, blurred vision, or floaters can be detected in patients with uveitis. Anterior uveitis is characterized by anterior chamber cells and flare, keratic precipitates, posterior synechiae, iris nodules, and cataracts. The clinical features of intermediate uveitis include grey-white fibrovascular plaques (snowbanks), the presence of cells suspended in the vitreous, vitreous haze, and inflammatory aggregates within the vitreous. In contrast, the characteristics of posterior uveitis include lesions within the retina or choroid, commonly known as white spots. All clinical features of the above-mentioned three types of uveitis were revealed in panuveitis (**Table 3**). Moreover, Vogt-Koyanagi-Harada disease is a common ocular irAE associated with ICIs (45, 58, 105). It is a type of bilateral granulomatous uveitis, associated with exudative retinal detachment and extraocular manifestations, such as pleocytosis in the cerebrospinal fluid and, in some cases, vitiligo, poliosis, alopecia, and dysacusis.

The majority of previously described uveitis on ICI therapy exhibited relatively mild to moderate severity, with ≤2+ anterior chamber cells and vitreous cells (28, 30, 77). In our review, 16 patients with uveitis and six patients did not manifest the detailed clinical features. Among the remaining patients with lung cancer, 70.00% were classified as grade 3 with anterior uveitis, comprising ≥3+ cells or intermediate posterior or panuveitis, based on the Common Terminology Criteria for Adverse Events (Version 5.0). While a total of 20.00% were classified as grade 4, only 10.00% were classified as grade 1 (**Table 2**). The average onset days of uveitis was 34.50 days on ICIs (**Tables 2**, **3**). There were 41.67% male patients, and 33.33% patients were older than 65 years. Moreover, 83.33% cases were unilateral.

Ocular examination including slit-lamp examination, ultrasound biomicroscopy, optical coherence tomography (OCT), ophthalmoscopy, fluorescein angiography or indocyanine green angiography are adapted for the diagnosis of uveitis. Diagnosis could be made based on clinical evidence including the clinical features and positive signs for auxiliary examination. Uveitis in lung cancer secondary to ICIs need to be differentiated from other disorder which might to presents as uveitis, including: infectious uveitis due to tuberculosis, syphilis or toxoplasma or other bacteria, autoimmune related uveitis, masquerade uveitis (105) (**Table 3**).

#### Dry Eye in Lung Cancer Secondary to ICIs

Dry eye disease is a multifactorial disorder of the tears and ocular surface, that caused by tear deficiency or excessive tear evaporation (106). It has been classified as dry eye with reduced tear production (occupying approximately 10%) and dry eye with increased evaporation of the tear film (hyperevaporative disorders) (107). Dysfunction of the meibomian glands is the primary cause of the hyperevaporative disorders and occupied more than 80% of the patients with dry eye (108). Dryness, redness, fatigue, photophobia, a sensation of burning, stinging or foreign body or pruritus could be detected. Pronounced conjunctival redness and punctate epithelial erosions of the cornea are typical clinical manifestations of dry eye (107). Inflammation of the lid margin or meibomian glands could be detected in dry eye caused by hyperevaporative disorders. In addition, dry eye could be one of the manifestations of systemic syndrome, such as Sjögren’s syndrome. Sjögren’s syndrome is an intractable autoimmune disease, characterized by dry eye, dry mouth, and extra glandular syndrome (109). In this review, patients with sjögren’s syndrome consisted of 92.86% of dry eye following ICI in lung cancer.

A comprehensive history (symptoms, systemic diseases and medication history), tear film break-up time with fluorescein, schirmer test, examination of the eyelid margins and meibomian gland orifices with expression of meibomian secretion could be conducted based on the diagnostic guidelines were published in 2007 by the Dry Eye Workshop (107, 110). In addition, screening for autoimmune diseases should be done as well, especially for Sjögren syndrome (111). The Gum test, the unstimulated whole saliva, saxon test, the labial salivary glands biopsy, and parotid glands biopsy are helpful for the diagnosis of Sjögren syndrome (109).

#### Conjunctivitis

Conjunctivitis caused by ICIs are the inflammation of conjunctiva which covers the inner surface of the eyelids and the white part of the eyeball. The blood vessels are enlarged and become more prominent in conjunctivitis. Red eye is the most common signs of conjunctivitis. Itchy, watery, burning or stinging eye and foreign-body sensation could be detected in patients with conjunctivitis. Ophthalmologist could give a diagnosis of conjunctivitis based on the Slit-lamp examination. Sodium hyaluronate, antihistamine eye drops or topical corticosteroids can help with symptoms of conjunctivitis after use of ICIs.

#### Corneal Perforation

Corneal perforation is the thinning and perforation of the cornea. Red eyes, severe pain, foreign-body sensation, tears, thick discharge, blurry vision, pain when looking at bright lights, swollen eyelids, and a white round spot on the cornea that is visible to the naked eye. The classic signs are shallowing or flattening of the anterior chamber, aqueous leakage, brown pigment from the iris in the wound could be detected. For the treatment of the corneal perforation, the first step is to discontinue the ICIs (112). Medical treatment is the second therapeutic step, including artificial tear drops, corticosteroids and cyclosporine. Timely diagnosis and prompt medical treatment could improve the rate of the surgical success (62, 112). Several surgical strategies could be used and it depends on the size, position, and depth of the ulceration (112, 113). The surgical management of corneal perforation includes corneal gluing, Collagen cross-linking with photo-activated riboflavin, Amniotic membrane transplantation, Conjunctival flap transplantation, Corneal transplantation.

#### Corneal Graft Rejection

Corneal graft rejection is a complex immune-mediated response, which leads to corneal graft decompensation (114). The rejection can occur in all of the layers of the cornea (epithelium, stroma and endothelium). Pain, redness, and decreased vision could be present in patients suffering corneal graft rejection. Conjunctival hyperemia, keratic precipitates, opacity and edema of corneal graft could be detected. It is not difficult to give diagnosis of corneal graft rejection based on the slit-lamp microscope. Prevention, early detection, and rapid management are crucial for the management of graft rejection (114, 115). Stop the ICIs is the essential which have been recommended in the previous study (41). Corticosteroids (Topical and systemic corticosteroids, intravenous pulsed corticosteroid therapy), cytotoxic agents (azathioprine), cyclosporin A have been used for management of corneal graft rejection (114).

#### Retinopathy

Retinopathy after use of ICIs might be caused by abnormal cross-reactivity of autoantibodies directed to retinal antigens. Vision loss, scotomas, photopsia, nyctalopia could be found in patients with retinopathy (116). Optical coherence tomography, fundus autofluorescence, visual field and electrophysiology could help us to detect to lesion on the retina. Medical history and physical exam findings are important for us to determine the risk factors of immune related retinopathy. High suspicion and early diagnosis and treatment are essential to reduce the risk of irreversible immune damage to retinal cells. Systemic and/or topical corticosteroids, immunomodulators (cyclosporine, infliximab, et al), biologics (rituximab, alemtuzumab, et al), intravenous immunoglobulin (IVIG) and plasmapheresis have been advocated for the treatment of immune related retinopathy (116–118).

#### Optic Neuritis

Typically, optic neuritis is unilateral. Eye pain, vison loss, the loss of the visual field, flashing lights could be detected in patients with optic neuritis. Ocular examination including slit-lamp examination, pupillary light reaction test, optical coherence tomography, visual field test, visual evoked response is adapted for the diagnosis of optic neuritis (119, 120). High-dose corticosteroids is effective for the treatment of optic neuritis. For the steroid-resistant optic neuritis, plasma exchange is needed (119).

#### Amaurosis Fugax

Amaurosis fugax refers to transient visual loss caused by the temporary ceasing of the retinal blood flow (121, 122). The time of amaurosis fugax could be last 2-30 minutes. Hypoperfusion, vasospasm, thromboembolism from a carotid plaque, elevated plasma viscosity and cerebrovascular disease could be pathogenic causes of amaurosis fugax (121). Comprehensive ocular examination and assessment of cardiovascular system is essential. An electrocardiogram, Magnetic resonance angiography, blood test and so on should be performed. The primary goal of treatment is to control and treat potential vascular risk factors (121).

#### Giant Cell Arteritis

Giant cell arteritis is primary vasculitis which mostly invades large vessels. The clinical characteristics is with strong heterogeneity, the common systemic manifestations are headaches, scalp tenderness, jaw claudication, vision loss, absent pulses and limb claudication (123, 124). About two-thirds patients could be detected ocular symptoms. Blurred vision is the most common manifestations (125). Diplopia, amaurosis fugax and blindness could be also present. Comprehensive ocular examination including ophthalmoscopy, FFA and ICGA are needed. Additionally, biopsy of the temporal artery, high-resolution color doppler ultrasound of the cranial and axillary arteries, MRI, CT scan need to be recommended for the diagnosis of Giant cell arteritis. Glucocorticoids has been considered as the primary treatment for Giant cell arteritis. Tocilizumab is also been approved by the FDA (124).

#### Orbital Inflammation

Orbital inflammation is characterized by infiltration of inflammatory cells, which is confined to the orbit, but may extend to the extraorbital area. Categories of orbital inflammation include dacryoadenitis, myositis, perineuritis of the optic nerve, periscleritis, diffuse sclerosing inflammation, and orbital apex inflammation. Eye pain, proptosis, decreased visual acuity, and eye movement restriction that may result in diplopia were the most common symptoms. Obvious orbital masses can be found by radiologic examination (126). Laboratory evaluation, Orbital ultrasound, Computed Tomography (CT), and Magnetic Resonance Imaging (MRI) may aid in the diagnosis when combined with clinical findings (127). Current therapeutic methods available for orbital inflammation include corticosteroids, nonspecific steroid-sparing agents(methotrexate, cyclosporin-A et al), biologic agents (infliximab, adalimumab and so on) and radiation therapy (128).

## Contributory Factors of Ocular irAEs in Lung Cancer Secondary to ICIs

### Ethnicity

Asian and Caucasian patients with lung cancer have different epidemiology, molecular profiles, and genetic susceptibilities (129, 130). Different incidences of irAEs secondary to ICIs could be detected between Asian populations and Western populations. The irAEs of grades 3–5 also present different prevalence rates between Asian and Western populations (131). Moreover, researchers could also detect differences in ocular irAEs associated with ICIs. A review of the IRIS Registry reported on a higher frequency of ocular irAEs in the Black population (9.7%, six of 62 patients) than that in the White population (3.5%, 91 of 2623 patients) (30). In addition, the Black population demonstrated a higher rate of ICI-related uveitis than their White counterparts.

In this review, among all patients with lung cancer and ocular side effects, 68.12% and 31.88% were Caucasians and Asians, respectively. The majority of patients with ICI-related ocular irAEs were reported in America (42.03%) and Japan (26.09%). The incidence of ophthalmoplegia was 43.75% (14/32) in Asians, compared to 12.77% (6/47) in Caucasians (**Table 2**, **Figure 3**, and **Figure S1**). Based on the multivariate Cox regression analysis, ethnicity was presented as an important factor that influenced ocular irAEs (**Figure 3**). Ophthalmoplegia was more frequently detected in Asians than in Caucasians (**Table 2** and **Figure 3**). However, no significant difference has been detected in the onset time of ocular irAEs in lung cancer (**Figure S1**). Thus, ethnicity could be an important factor in the type of ocular irAEs following ICI use.

### Types of ICIs

CTLA-4 inhibitors are reportedly associated with a higher frequency of irAEs and distinct profiles, compared to PD-1 inhibitors (132, 133). Moreover, the proportion of grade 3-4 irAEs is higher with CTLA-4 inhibitors (31%), compared to PD-1 inhibitors (10%) (132, 134). Data from a recent clinical trial reported on lower overall incidence of AEs in monotherapy with ICIs than that of combination therapy in NSCLC (89, 135). Furthermore, PD-L1 inhibitors combined with chemotherapy have a higher incidence of irAEs than monotherapy with PD-L1 inhibitors (98.2% *vs*. 70.9%, respectively) in NSCLC (92, 116, 117). Researchers have also identified differences in the distribution and incidence of ocular irAEs. Ocular surface adverse effects occur more frequently with PD-L1 (31). Uveitis is more likely to occur in patients following ICI therapy with CTLA-4 inhibition than in those with PD1 inhibition (14, 30, 136). In addition, ocular myasthenia reveals the highest association with nivolumab, followed by pembrolizumab (136).

Based on the reported ICI-related ocular irAEs, 44.30% of the patients with lung cancer were treated with PD-L1 inhibitors. In contrast, 36.71% and 11.39% were treated with PD-1 inhibitors and PD-L1 plus CTLA4 inhibitors, respectively. There was no significant difference in the distribution of ocular irAEs between PD-L1 inhibitors and PD-1 inhibitors. Nonetheless, significant differences were detected between monotherapy (PD-L1/PD-1 inhibitors) and combined therapy (PD-L1 plus CTLA4 inhibitors) (**Table 2** and **Figure 3**). Based on the multivariate Cox regression analysis, combined therapy was significantly more prone to ophthalmoplegia than monotherapy (**Figure 3**). The average onset time of ICI-related ocular irAEs with combined therapy with PD-L1 plus CTLA4 inhibitors (6.98 weeks) was shorter than that in patients treated with PD-1 (8.88 weeks) and PD-L1 inhibitors (17.47 weeks). However, the difference was insignificant (**Figure S1**).

### Pre-Existing Disorders

Pre-existing disorders are the most important risk factors for ICI-induced irAEs (137, 138). Moreover, 27% of the patients with a history of autoimmune diseases could suffer from exacerbations of the autoimmune condition, which requires systemic treatment following the use of ICIs (30). With a history of non-ophthalmic autoimmune diseases, ocular irAEs could be detected in 27-40% patients undergoing ICI treatment (30, 139). The incidence of ICI-related uveitis could be as high as 51.10% in patients with prior uveitis diagnosis, and up to 36.40% of patients experience various neuro-ophthalmic complications (139). In addition, approximately 20.00% of the patients with Sicca/Sjögren’s syndrome following the use of PD-1/PD-L1 checkpoint inhibitors reportedly have a history of previous autoimmune diseases (personal or familial), thereby indicating a predisposing immunogenetic background, according to the data from the International Immuno Cancer Registry (ICIR) (24). In this review, one patient with lung cancer reported a history of inactive uveitis. Following ICI use for 2 months, uveitis with 2+ anterior chamber cells and fine keratic precipitates were detected in both eyes (43). Therefore, pre-existing autoimmune diseases could play a non-negligible role in the occurrence of ICI-related ocular irAEs, thus warranting more attention to medical history.

### Other Factors

#### Age

According to a retrospective study, patients older than 70 years demonstrated comparable efficacy and safety outcomes for ICIs than younger patients (140). Better long-term outcomes were detected in older patients (140, 141). Furthermore, irAEs followed by ICIs had similar efficacy outcomes. Grade 3–4 irAEs rates did not reveal statistical differences between older (11%, ≥70 years) and younger patients (12%, <70 years) (142, 143). In this review, the mean age at the time of ocular irAE diagnosis in patients with lung cancer was 66.84 ± 10.36 years. Based on the multivariate Cox regression analysis, the age was not an influencing factor for ocular irAEs (p= 0.37). However, patients in pivotal clinical trials were commonly selected, particularly older patients with ICIs as are frailer (144, 145). In addition, there are limited reports on ocular irAEs in lung cancer, therefore necessitating further evaluation of the efficacy and safety of ICIs for older patients in a real-life setting (**Figure 3** and **Figure S1**). Moreover, the onset time of ocular irAEs is not related to the age of patients with lung cancer.

#### Gender

Throughout the course of life, the incidence of malignancy is higher in men than women (146). However, cancer treatments in men have also demonstrated significantly better outcomes than those in women. Gender is a reportedly relevant element that modulates the expression of the PD-1 pathway (147). In addition, male patients demonstrate a better efficacy of single agent ICIs treatment than their female counterparts (147, 148). No studies have illustrated the difference in ICI-related irAEs between men and women (146, 149). Considering the vulnerability of women to autoimmune responses, the frequency of irAEs following ICIs might be more likely to occur in women than in men. In this review, females accounted for 39.13% of the patients with ocular irAEs in lung cancer. Moreover, we detected no significant gender difference among patients with ophthalmoplegia and other ocular irAEs, based on the multivariate Cox regression (**Table 2**, **Figure 3**, and **Figure S1**).

#### Types of Tumor

Different tumor types may cause different irAEs following ICIs. In a previous review involving 6938 patients with different tumor types, melanoma showed a higher incidence of gastrointestinal and skin irAE and lower incidence of pneumonitis after use of ICIs (132). In general, NSCLC represents 85% of all lung tumors, and the other 15% is SCLC (150, 151). In a review involving 14256 patients with lung cancer, it concluded that the incidence of ICI-related irAEs in individuals with NSCLC is less than with SCLC (21). While in this review, only three patients with SCLC suffered ocular irAEs. No studies with a large sample size of individuals focus on the ocular irAEs are reported and we cannot conclude the difference of the incidence of ocular irAEs between SCLC and NSCLC (21).

## Management Strategies and Outcomes for Ocular irAEs

For the treatment of the ocular irAEs following ICIs, almost all cases of ocular irAEs were managed with conservative treatment, including topical or periocular corticosteroids. Symptomatic treatment is essential for controlling ocular irAEs, such as topical sodium hyaluronate for dry eye and cyclosporine for corneal perforation (152–154). Systemic treatment and suspension of ICIs were used in uncontrolled and serious cases, such as corneal graft rejection, corneal perforation. Based on the recommended guidelines of the ocular irAEs. The management and outcome of ophthalmoplegia, uveitis and dry eye had been described in detail as follows. Other ocular irAEs have been simply described clinical manifestation of ocular irAEs in part 3.

### Management Strategies and Outcomes for Ophthalmoplegia

**Figure 4:**
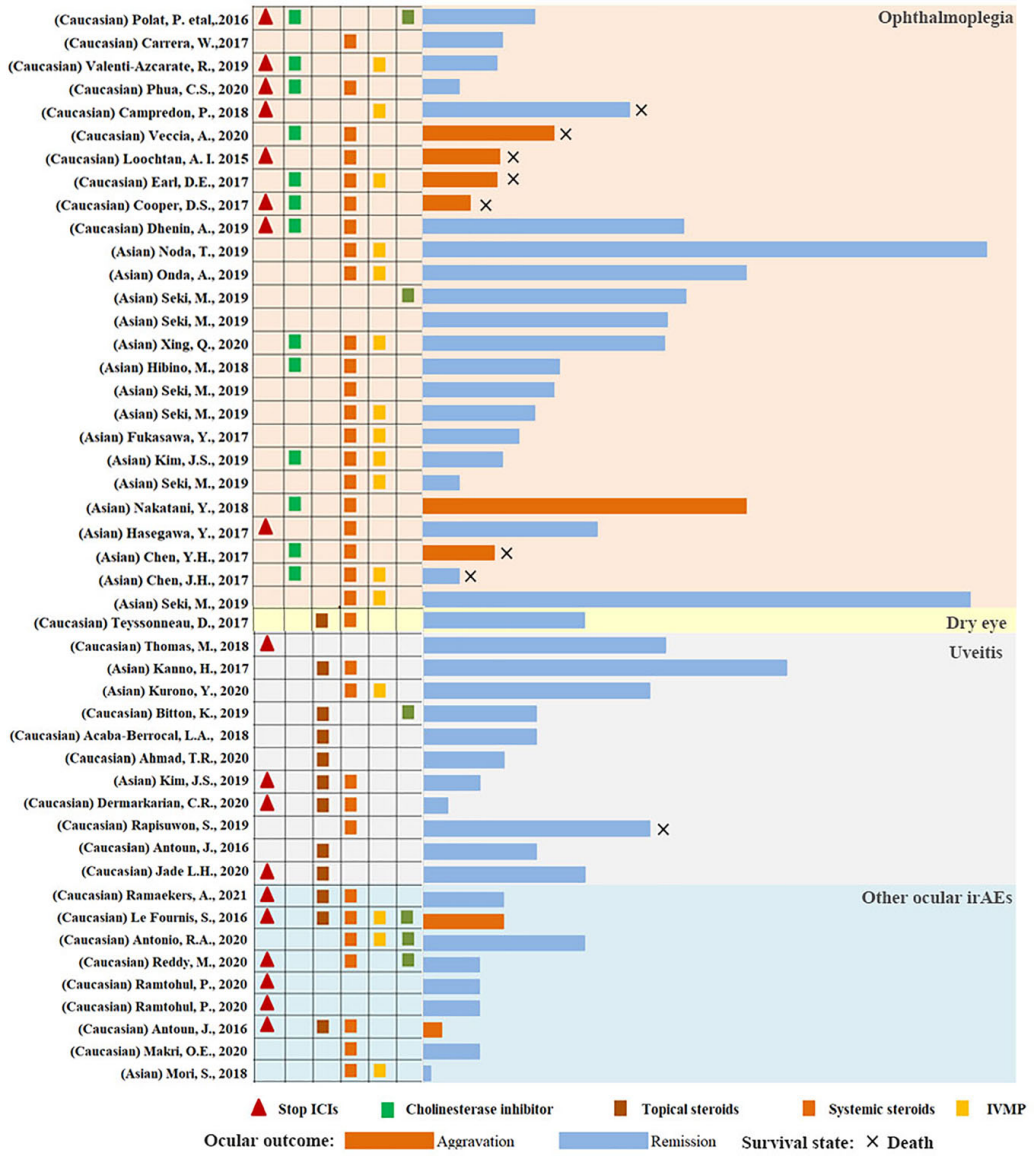
The course of the ocular irAEs following ICI use in lung cancer. The column indicates the length of the complication in each patient with ocular irAEs. Light pink, light gray, light yellow, and light blue represent ophthalmoplegia, uveitis, dry eye, and other ocular irAEs. The blue column represents remission or complete recovery. The dark yellow column represents an aggravation of disease or death.

Cholinesterase inhibitors (pyridostigmine) are the mainstay of therapy for ophthalmoplegia in MG. They are quick, safe, and free of long-term side effects (155). However, corticosteroids are required if cholinesterase inhibitors produce no response. A randomized controlled trial compared prednisone and placebo in patients with ocular MG who had previously failed to achieve minimal manifestation status, following 4 to 6 weeks of pyridostigmine use. Eighty-three percent of the patients under prednisone treatment acquired faster and better remission than those receiving placebo (156). Corticosteroids are widely available and cheap, and are the next step of treatment. They reportedly reduce the rate of generalization in patients with ptosis in MG (157). Low-dose corticosteroids might be more effective for ptosis in MG, and may decrease side effects with high-dose corticosteroids. Therapy with immunosuppressive and intravenous immunoglobulin or plasmapheresis have been found effective in a cohort of patients with MG (93, 94). It could also be used in patients treated with corticosteroids who were still symptomatic or had contraindications to corticosteroids, and experienced severe side effects with advanced systemic affections (89, 158). Suspending ICIs therapy is not necessary for the treatment of ophthalmoplegia in severe autoimmune MG (159). In this review, 84% of ocular irAEs in lung cancer followed by ICIs could acquire complete clinical recovery (**Table 2** and **Figure 4**). The rate of ophthalmoplegia aggravation (23.33%) was significantly higher than that of other ocular irAEs (7.69%). In addition, the mortality of patients with ICI-related ophthalmoplegia was higher in lung cancer as well.

### Management Strategies and Outcomes for Uveitis

Therapies for ICI-induced uveitis focus on controlling inflammation and decreasing the frequency of recurrence. Mydriasis prevents the formation of iris adhesions. Moreover, it can relieve photophobia from iris sphincter spasm and the pain of ciliary muscle action associated with iridocyclitis. Topical corticosteroids and systemic corticosteroids are the mainstays of treatment. The probability of uveitis relapse necessitates the maintenance of corticosteroids for patients who continue ICI therapy (160). Topical corticosteroids are usually effective in controlling inflammation in anterior uveitis. However, systemic corticosteroids are required for severe anterior uveitis, posterior uveitis, or panuveitis in lung cancer following ICI use (161). In addition, subconjunctival corticosteroids, intravitreal dexamethasone implant, and triamcinolone periocular space injection could also be effective. Uveitis detection might not be a sign to suspend ICI therapy, as the majority of ocular irAEs could acquire an excellent and rapid response to conventional treatment, with generally favorable clinical outcomes (28). In our review, all patients with uveitis in lung cancer following ICI use could be remised or acquired complete clinical recovery. Moreover, the average time of remission was 62.82 ± 38.48 days (**Table 2** and **Figure 4**), consistent to previous studies (28, 32, 88).

### Management Strategies and Outcomes for Dry Eye

Preservative-free artificial tears are the mainstay of therapy for all severity grades of dry eye, which could increase tear film stability, improve contrast sensitivity and the optical quality of the surface. Autologous serum eyedrops could be useful and apply in in severe cases of dry eye. In addition, anti-inflammatory treatment should be conducted in moderate to severe cases with dry eye. Topical corticosteroid eyedrops for 2 to 4 weeks had been reported symptomatic improvement in a randomized and double-masked study (162). Cyclosporine A could increase the production of tear fluid, and had been reported to reduce symptoms, improve the Schirmer test values in previous studies (109, 111, 163). It had been approved by FDA for treatment of dry eye. However, systemic corticosteroid, immunosuppression or suspension of ICIs are not recommended for dry eye (109).

## Limitations

There are several limitations in this review. At first, the sample size is limited. Only 79 patients with ocular irAEs in lung cancer had been searched. Most of the recruited cases are from case report or case series, we cannot deduce the accurate incidence of the ocular irAEs in lung cancer following ICIs. Moreover, some ocular irAEs with a lower frequency might not be reported. Secondly, most of studies were focused on the systemic irAEs not the ocular irAEs and the detailed clinical characteristics of the ocular irAEs are not available. The treatment of the ocular irAEs in different studies were not identical as well, including the initiation time and dose of the drugs, the types of drugs, following time and so on. We do not summarize the detailed features and treatment of each ocular irAEs based on the recruited studies.

## Conclusions

ICIs have greatly changed the prognosis of lung cancer, which was previously considered as a fatal tumor. With the widespread use of ICIs, more and more related toxicities have been reported. Although ocular irAEs are infrequent based on the previous study, they can cause a deterioration of the quality of life and exert an influence on the compliance of patients. Lots of studies have reported the ocular adverse events secondary to ICIs (19, 25, 26, 30, 31, 153, 164–168) and the grade of the adverse events had been published recently based on Common Terminology Criteria for Adverse Events. While no study had reported the ocular irAEs in lung cancer. Previously, dry eye and uveitis were the most common ocular irAEs. However, ophthalmoplegia especially ptosis, has been considered as the most common reported irAEs in lung cancer in this study.

All of the patients with ophthalmoplegia secondary to ICIs are the complication of myasthenia gravis in this study. While the most fatal manifestations including respiratory depression and myocarditis can be detected in approximately two-thirds of individuals with myasthenia gravis. The high incidence of ophthalmoplegia with myasthenia gravis in ocular irAEs and the high mortality of life-threatening myasthenia gravis in lung cancer necessitate an increase in ophthalmoplegia vigilance. This reminds us of timely identification of the ophthalmoplegia with myasthenia gravis, particularly in the early stages of irAEs. Based on this study, we found that the prevalence of ophthalmoplegia in Asian, the combination therapy of PD-L1+CTLA4 inhibitors were significantly higher than uveitis or other ocular irAEs. Pre-exiting autoimmune diseases could cause a higher incidence of the ocular irAEs in lung cancer. The onset time of the ophthalmoplegia is earlier than other ocular irAEs (within 10 weeks after initiation of ICIs). This could help us to easily diagnose and identify the ocular irAEs, especially for ophthalmoplegia.

Due to the sample size of ocular irAEs in lung cancer is limited and most of the recruited patients were come from case reports, further additionally studies on ocular irAEs were urgently needed to illustrate the ICI-related ocular irAEs. The understanding of ocular irAEs is necessary to guide the proper prevention and treatment plan and improve the quality of life of patients. Open communication between internist, oncologist and ophthalmologists is necessary to identify and manage the ocular irAEs.

## Author Contributions

LZ searched the literature and wrote the manuscript and conducted the statistics analysis. XW revised the manuscript, and verified the study. All authors contributed to the article and approved the submitted version.

## Funding

Supported by grants from the Natural Science Foundation of China (No. 82070954); The Applied Basic Research Programs of Science and Technology Commission Foundation of Sichuan Province (No. 19YYJC0790); The Innovative Spark Grant of Sichuan University (No. 2018SCUH0062).

## Conflict of Interest

The authors declare that the research was conducted in the absence of any commercial or financial relationships that could be construed as a potential conflict of interest.

## Publisher’s Note

All claims expressed in this article are solely those of the authors and do not necessarily represent those of their affiliated organizations, or those of the publisher, the editors and the reviewers. Any product that may be evaluated in this article, or claim that may be made by its manufacturer, is not guaranteed or endorsed by the publisher.
