# Supplementary figures and images for: Ocular Immune-Related Adverse Events Associated With Immune Checkpoint Inhibitors in Lung Cancer
Source: Front Immunol. 2021 Aug 24;12:701951. doi: 10.3389/fimmu.2021.701951 (PMC8421677; doi:10.3389/fimmu.2021.701951)

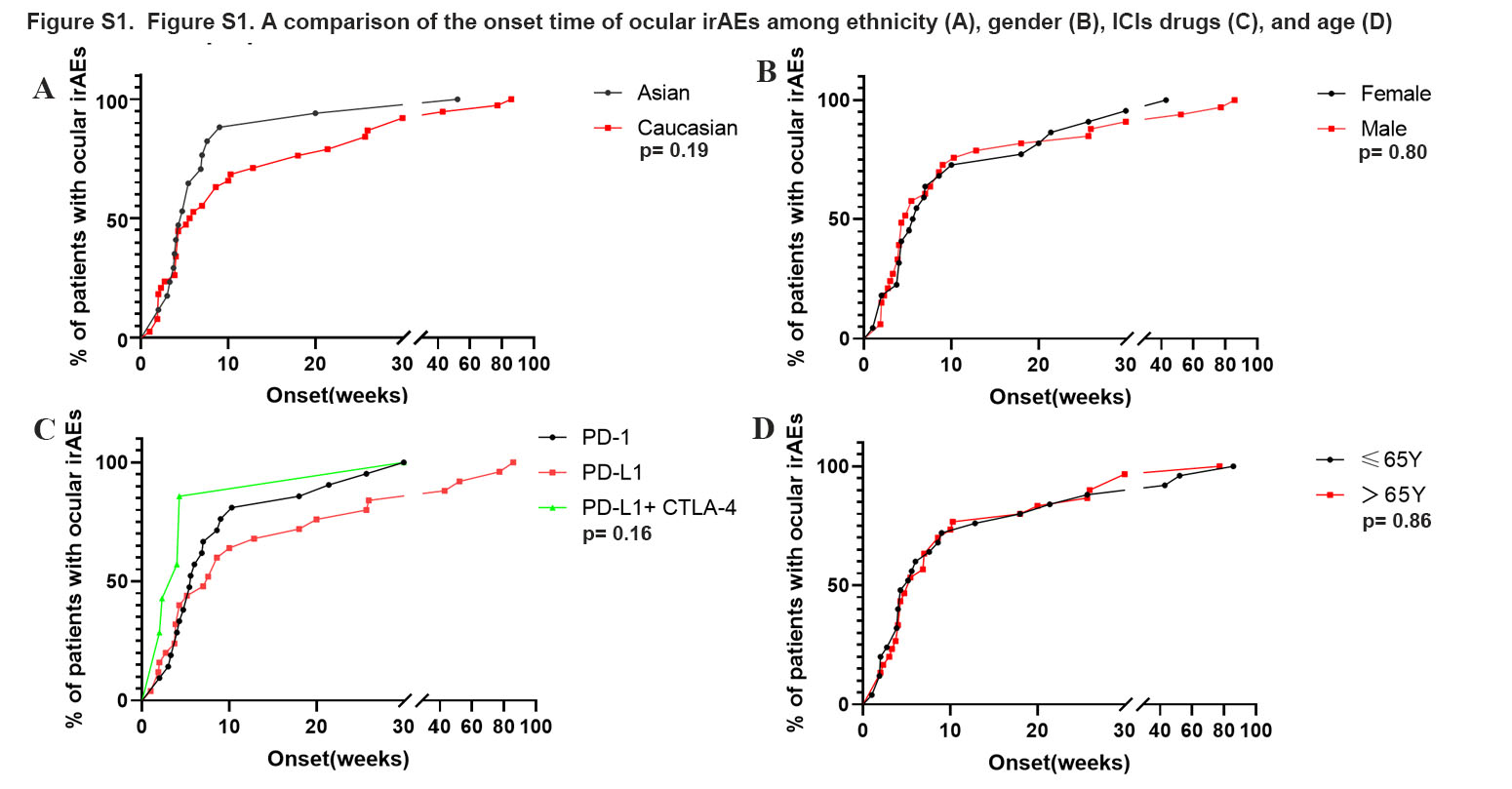

Supplement: Supplementary file 1 [file Image_1.jpg]
